# Preparation and Characterization of an Ancient Aminopeptidase Obtained from Ancestral Sequence Reconstruction for L-Carnosine Synthesis

**DOI:** 10.3390/molecules27196620

**Published:** 2022-10-05

**Authors:** Fan Liu, Yi Shi, Yakun Fang, Zhenshan Liu, Yu Xin, Zhenghua Gu, Zitao Guo, Liang Zhang

**Affiliations:** 1 National Engineering Research Center of Cereal Fermentation and Food Biomanufacturing, Jiangnan University, Wuxi 214122, China; 2 Jiangsu Provincial Research Center for Bioactive Product Processing Technology, Jiangnan University, Wuxi 214122, China

**Keywords:** ancestral sequence reconstruction, aminopeptidase, peptide synthesis, heterologous expression, pH tolerance

## Abstract

As a biologically active peptide, L-carnosine has been widely used in the pharmaceutical, cosmetic and health care industries due to its various physiological properties. However, relatively little research is available regarding L-carnosine’s enzymatic synthesis function. In this study, a potential enzyme sequence with the function of carnosine synthesizing was screened out using the ancestral sequence reconstruction (ASR) technique. Identified with L-carnosine synthesis activity, this enzyme was further confirmed using autoproteolytic phenomenon via Western blot and N-terminal sequencing. After purification, the enzymatic properties of LUCA–DmpA were characterized. The melting temperature (*T_m_*) and denaturation enthalpy (Δ*H*) of LUCA–DmpA were 60.27 ± 1.24 °C and 1306.00 ± 26.73 kJ·mol^−1^, respectively. Circular dichroism (CD) spectroscopy results showed that this ancestral enzyme was composed of α-helix (35.23 ± 0.06%), β-sheet (11.06 ± 0.06%), β-turn (23.67 ± 0.06%) and random coil (32.03 ± 0.06%). The enzyme was characterized with the optimal temperature and pH of 45 °C and 9.0, respectively. Notably, LUCA–DmpA was also characterized with remarkable pH tolerance based on the observation of more than 85% remaining enzymatic activity after incubation at different pH buffers (pH = 6–11) for 12 h. Additionally, rather than being improved or inhibited by metal ions, its enzymatic activity was found to be promoted by introducing organic solvent with a larger log *P* value. Based on these homology modeling results, the screened LUCA–DmpA is suggested to have further optimization potential, and thereafter to be offered as a promising candidate for real industrial applications.

## 1. Introduction

Firstly discovered in beef in 1900 by a Russian chemist, L-carnosine is a bioactive β α-dipeptide composed of β-alanine (β-Ala) and L-histidine (L-His) [1]. As a substance widely distributed in mammalian skeletal muscles, brain and other tissues, carnosine in different tissues was reported to have the outstanding property of anti-oxidation [2]. As a result, L-carnosine has been implemented to serve as the main component in anti-oxidant, anti-glycation, anti-cancer, and anti-aging substances [3,4,5]. In addition, carnosine plays an effective role in aspects of intracellular pH buffering, chelation of metal ions, neuromodulation and radical scavenging. L-carnosine has been widely applied in the food additive, health-care, cosmetic and medicine fields, which take advantage of its excellent antioxidant and various physiological properties [6,7,8,9]. Three methods to obtain L-carnosine, including chemical synthesis, extraction from animal tissues and biosynthesis, have been reported [10,11,12]. Although chemical synthesis is extensively reported as a commercial method to produce L-carnosine at present, complex reaction processes and conditions, highly toxic reagents and high-energy consumption make it environmentally unfriendly, thus highlighting the requirement for other simple and environmentally friendly synthesis pathways [13]. In comparision, enzymatic synthesis of dipeptides has been revealed as a promising solution due to its gentle reaction conditions and environmental friendless, whereas aminopeptidases are commonly reported with dipeptides’ synthetization function. 

With the capability to selectively hydrolyze N-terminal amino acid residues of polypeptides and proteins, belonging to the exopeptidases, aminopeptidases are found to be widely distributed in animals, plants and microorganisms [14]; microbial aminopeptidases (EC 3.4.11) found in prokaryotes and eukaryotes are considered one of the earliest discovered proteases [15]. Based on its catalytic mechanism, substrate specificity or molecular structure, aminopeptidase could be classified into different categories. Although their catalytic mechanisms vary, many peptidases are Zn^2+^-dependent metalloenzymes [16]. Except for catalyzing in hydrolysis, promoting the synthesis of β and mixed α, β-peptidases was also determined to be characteristic of certain aminopeptidases [17]. Regarding the function of synthesis, particularly for L-carnosine, the highest reported L-carnosine concentration was 19.3 mM [18]. In addition, aminopeptidases with L-carnosine synthesis activity, including the BapA from *Sphingosinicella xenopeptidilytica* and the DmpA from *Ochrobactrum anthropic*, were all classified into the N-terminal nucleophile hydrolases family [19,20], thus suggesting a potential clue to the screening of sequences with better L-carnosine synthesis efficiency using methods such as ASR. 

ASR is a powerful computational method for predicting ancient protein sequences from extinct species based on the sequences of modern organisms [21]. Calculating potential ancestral protein sequences existing in all nodes of the phylogenetic tree has been revealed as a powerful method for probing evolutionary relationships and designing proteins with more desirable properties. Generally, the main procedures performed using ASR to simulate the natural evolutionary trend of the target protein are target protein homologous sequences selection, homologous present-day proteins multiple sequence alignment (MSA), best amino acid substitution model selection and the maximum likelihood phylogenetic tree calculation [22]. Based on the predicted result, amino acids that may appear in the non-conserved regions of the target protein can be inferred [21]. In addition to the desired catalytic activity, previous studies suggested that predicted ancient proteins tend to be more thermostable, easier to express, and have a broader substrate spectrum when compared with present-day proteins [23,24,25]. Therefore, by taking advantage of ASR, it is possible herein to obtain a novel aminopeptidase with enzymatic properties more suited to the synthesis of L-carnosine by expressing predicted ancestral sequences via the above principles.

In this study, reported aminopeptidase protein sequences were collected to screen for potential novel aminopeptidase with the catalytic activity of L-carnosine synthetization. To further determine the application potential of the predicted protein, enzymatic properties including thermal stability and the tolerance of acidity, alkalinity, organic solvents and metal ions were investigated after obtaining purified enzyme generated from the expression products produced by *E.* *coli* BL21 (DE3). Relying on the ASR method and systematical analysis of promising novel predicted aminopeptidase enzymatic properties, the current study is expected to lay the foundation for the industrial application of this type of enzyme in the production of L-carnosine.

## 2. Results and Discussion

### 2.1. Ancestral Sequence Reconstruction of Aminopeptidase

**Figure 1:**
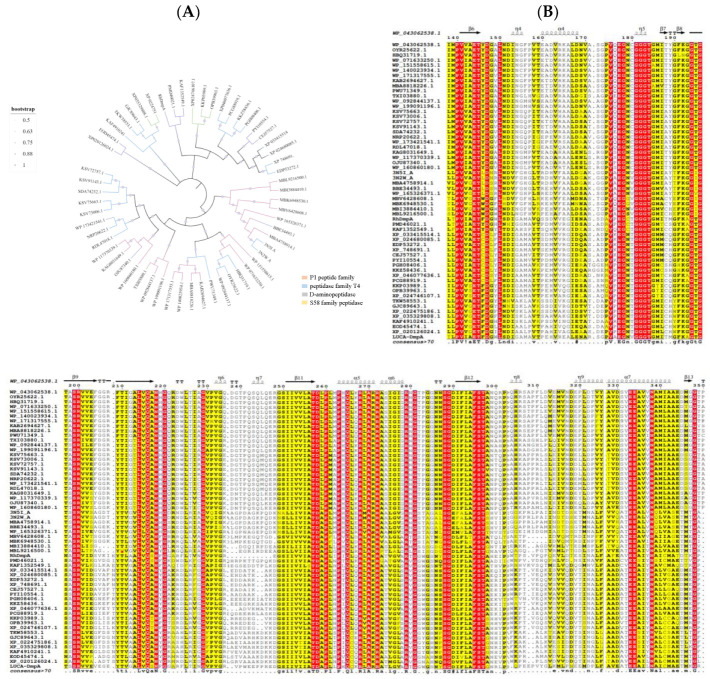
The maximum likelihood phylogenetic tree and multiple sequence alignments of protein sequences. (**A**) The phylogenetic tree was constructed via the maximum likelihood method using PhyML v 3.0. The simplified phylogenetic tree is presented as Supplementary Data (Figure S1). (**B**) Multiple sequence alignments of protein sequences performed using Clustal X 2.1 and visualized using ESPript 3.0 (WP_043062538.1 as template).

In the last few years, the ASR technique had been applied in the study of genes, proteins and the evolution of enzymes. A series of enzymes having more desirable characteristics, or new properties, were reconstructed from the ASR [21,26,27]. In this study, three sequences (XP-033415514.1, WP_043062538.1 and 3N5I-A) with the peptide synthesizing function were selected as the template for screening for aminopeptidase with better enzymatic properties. As shown in Figure 1A, the maximum likelihood phylogenetic tree was constructed using the screens of 56 sequences from four peptidase families (P1, T4, D-aminopeptidase and S58). Overall, 51 identical amino acids, mainly distributed in the region of β-sheet, were identified. These were analyzed using Clustal X 2.1 and Espript 3.0 and used the secondary structure of sequence WP_043062538.1 as a template; Figure 1B illustrates the conserved amino acids of peptidases from different families. Although most amino acids related to the active center (i.e., Glu144, Gln216, Asn218, Ser288 and Gly289) within different peptidases were highly conserved, amino acids Ala250 in β-aminopeptidase (PDB ID: 3N5I-A) from *Sphingosinicella xenopeptidilytica* was found to be replaced. In addition, the Tyr146 was found to be substituted by Leu, Cys, Phe, Trp or other amino acids in most of these protein sequences. Based on the above analysis, the common ancestral sequence (LUCA), named LUCA–DmpA, was identified, and its enzymatic properties were further investigated.

### 2.2. Cloning, Expression and Purification of LUCA–DmpA 

**Figure 2:**
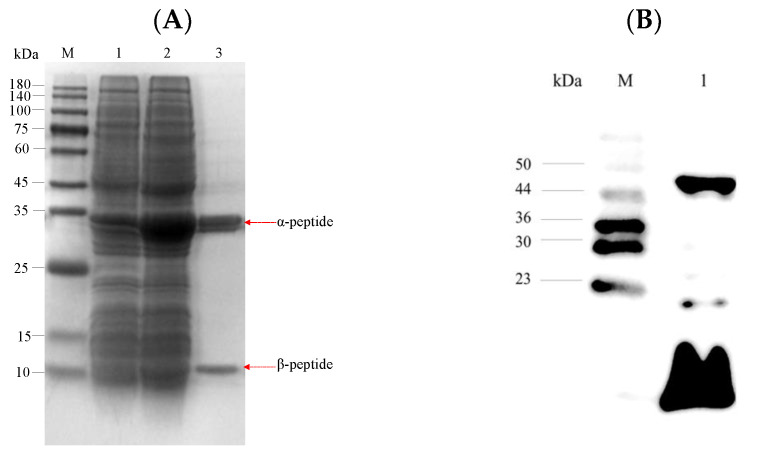
The induced expression of LUCA–DmpA and Western blot analysis. (**A**) SDS-PAGE analysis of LUCA–DmpA only containing N-terminal His-tag. Lane M, the protein marker; lane 1, control; lane 2, the crude enzyme of LUCA–DmpA; and lane 3, the purified LUCA–DmpA. (**B**) Western blot with an anti-His body performed with the purified LUCA–DmpA only containing a C-terminal His-tag. Lane M, the molecular marker of Western blot and lane 1, the purified LUCA–DmpA with C-terminal His-tag.

LUCA–DmpA was cloned into a pET28a(+) vector. After *E. coli* cell lysis, three overexpressed bands were observed in the crude extract via SDS-PAGE analysis of roughly ~42, 29 and 13 kDa when compared to molecular weight standards (Figure 2A). After purification, only the ~29 and ~13 kDa could be clearly seen. Western blot analysis identified that the ~40 and ~15 kDa bands contained a His-tag. This suggested that the protein with a predicted molecular weight of 42.1 kDa for the full length might self-cleave. N-terminal sequencing of β-peptide revealed the sequence of SIIVVIATDA for the 10 N-terminal residues. This result indicated that the mature aminopeptidase was cleaved at the Gly237-Ser238 peptide bond to form α and β peptide chains. Although unexpected, this autoproteolytic and catalytic mechanism of LUCA–DmpA was in accordance with the serine aminopeptidase DmpA from *Ochrobactrum anthropic* [16] and the β-aminopeptidase BapA from *Sphingosinicella xenopeptidilytica* [28]. This phenomenon might have been triggered by the requirement of peptide bond cleaving between Gly237−Ser238 to form α and β peptide chains before exerting its enzymatic activity. In addition, as the structure of Gly-Ser was conserved in most studied peptidase sequences, the autocatalytic formation of one-α and one-β subunits might then be indicated to be a typical feature of the studied peptidase family since time immemorial.

### 2.3. Characterization of LUCA–DmpA

**Figure 3:**
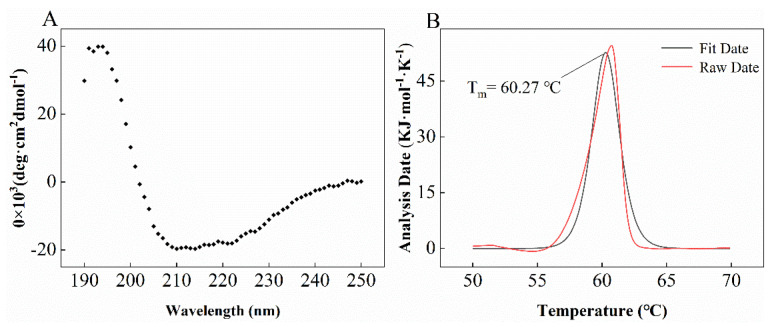
CD and nano-DSC assays. (**A**) The secondary structure of purified LUCA–DmpA contained α-helix (35.23 ± 0.06%), β-sheet (11.06 ± 0.06%), β-turn (23.67 ± 0.06%) and random coil (32.03 ± 0.06%). (**B**) Thermal denaturation midpoint temperature (*T_m_*) analysis of LUCA–DmpA was subjected to nano-DSC analysis; the *Tm* and Δ*H* of LUCA–DmpA were 60.27 ± 1.24 °C and 1306 ± 26.73 kJ·mol^−1^, respectively.

**Figure 4:**
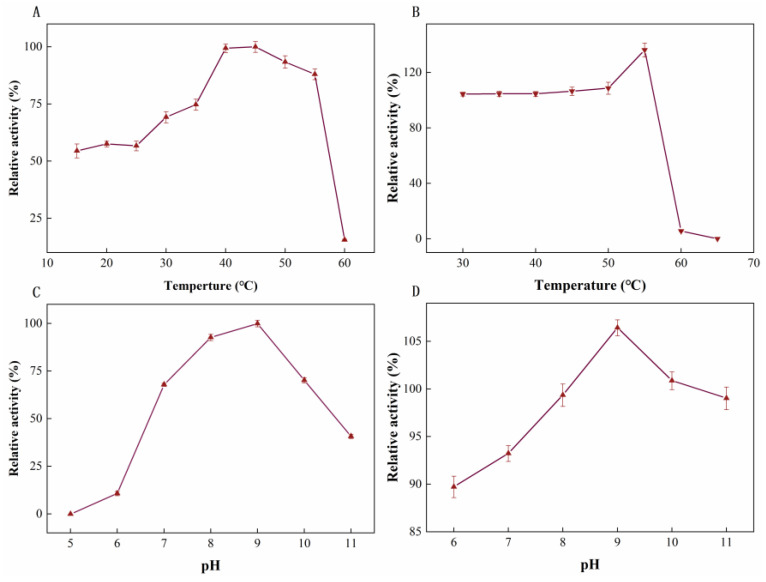
Effect of pH and temperature on LUCA–DmpA. (**A**) The optimum reaction temperature of LUCA–DmpA was 45 °C. (**B**) After incubation at 30–55 °C for 30 min, LUCA–DmpA ancient enzyme activity was stable; no remaining activity was detected when the temperature reached to 65 °C. (**C**) The optimum reaction pH of LUCA–DmpA was 9.0. (**D**) After LUCA–DmpA was incubated at different pHs (pH = 6–11) for 12 h, it showed higher relative activity.

To characterize the corresponding secondary structure, circular dichroism (CD) analysis results indicated that LUCA–DmpA was composed of α-helix (35.23 ± 0.06%), β-sheet (11.06 ± 0.06%), β-turn (23.67 ± 0.06%) and random coil (32.03 ± 0.06%) (Figure 3A). As shown in Figure 4B, nano-DSC analysis results revealed that the *T_m_* and Δ*H* of purified aminopeptidase were 60.27 ± 1.24 °C and 1306.00 ± 26.73 kJ·mol^−1^, respectively. 

### 2.4. Effects of pH and Temperature on Stability and Activity of LUCA–DmpA

As illustrated in Figure 4A, the optimal temperature of LUCA–DmpA was 45 °C; a decreasing trend in enzymatic activity was identified after the catalysis reacted at a temperature higher than 45 °C. This result was similar to present-proteins, such as D-aminopeptidase from *O. anthropi* SCRC CI-38, which was also identified with an optimum temperature of 45 °C [29]. When the temperature was increased to 60 °C, the remaining enzyme activity could only be detected as 10% by applying the maximum enzyme activity as 100%. In terms of thermostability (Figure 4B), after purified enzyme was incubated at 30–55 °C for 30 min, enzyme activity was higher than initial activity; only 5.60% relative activity remained after incubation at 60 °C for 30 min due to possible damage of the LUCA–DmpA conformation. The remaining activity could not be detected when the incubation temperature was increased to 65 °C, suggesting a consistency with the measured *T_m_* value. Compared with other reported aminopeptidases, such as BapA [30] from *Pseudomonas* sp., the current newly-expressed ancient enzyme tended to exhibit improved thermostability, as only 45% of the relative activity of BapA remained after incubation at 55 °C for 10 min.

Regarding the optimal pH of LUCA–DmpA, the enzyme showed a maximal activity at a pH of 9.0, thus indicating it was a type of alkaline aminopeptidase. After incubation at different pH conditions, over 85% of LUCA–DmpA’s initial activity (Figure 4D) was measured, indicating that LUCA–DmpA had a remarkable pH tolerance, which would stimulate promising industrial applications. 

### 2.5. Effect of Organic Solvents and Metal Ions on Enzyme Activity

**Figure 5:**
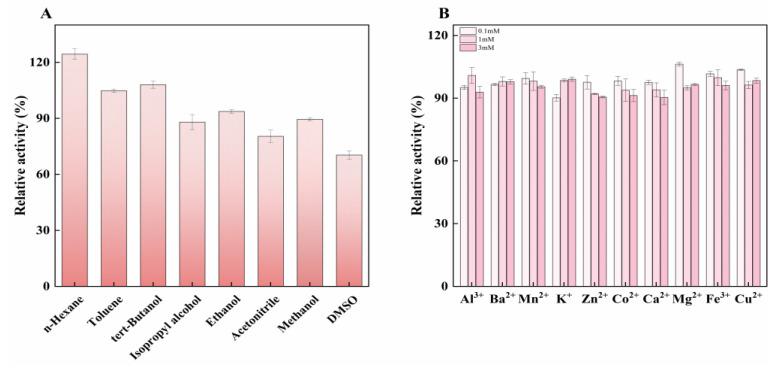
Effect of various organic solvents and metal ions on LUCA–DmpA. (**A**) LUCA–DmpA incubated with eight different organic solvents (50%, *v*/*v*) for 30 min. (**B**) Effect of different metal ions of varying concentrations (0.1 mM, 1 mM and 3 mM) on the enzyme activity of LUCA–DmpA.

The effect of organic solvents on enzyme activity was evaluated. The results revealed that the remaining relative enzyme activity tended to be higher when the enzyme was incubated with organic solvents with a higher log *P* value (Figure 5A). In detail, when compared with the control (enzyme activity measured without the addition of chemicals, 100%) in the presence of n-Hexane (log *P* = 3.50), toluene (log *P* = 2.50) and tret-butanol (log *P* = 0.78), the relative activity of LUCA–DmpA was found to be above 100%; the relative enzyme activity was 124.40% after incubation with n-Hexane. However, solvents with lower log *P* values, including isopropanol (log *P* = 0.05), ethanol (log *P* = −0.24), methanol (log *P* = −0.76), acetonitrile (log *P* = −0.33) and DMSO (log *P* = −1.3), led to a decrease in relative activity. DMSO showed the most significant inhibitory effect on the enzyme, resulting a 30% decrease in relative enzyme activity. Organic solvents with larger *log P* values studied herein might have favored the catalysis activity of LUCA–DmpA; this can be explained by the high proportion of non-polar amino acids (>50%) in the protein. Figure 5B illustrates the effect of three concentrations (0.1 mM, 1 mM and 3 mM) of various metal ions on the enzyme activity of LUCA–DmpA. The results showed that different concentrations of metal ions had no significant effect on the enzymatic activity of this recombinant enzyme. Regardless of the type of metal ion used, when the metal ion concentration reached 3 mM, the relative enzyme activity could be maintained above 90%, suggesting relative stability in response to metal ions additions. This characteristic differed from previously reported aminopeptidase with similar structures, such as D-aminopeptidase from *Ochrobactrum anthropic* [29] and BapA from *Pseudomonas* sp. [30], whose enzymatic activities were found to be inhibited to some extend by different metal ions, including Zn^2+^, Ag^+^ and Cd^+^.

### 2.6. Determination of Kinetic Parameters of LUCA–DmpA

In previous studies, aminopeptidase catalyzation for peptide synthesizing was suggested to be a kinetically controlled process [12]; most reports regarding aminopeptidases focused on their function in peptide hydrolysis, with limited reports about the ammonolysis function.

**Figure 6:**
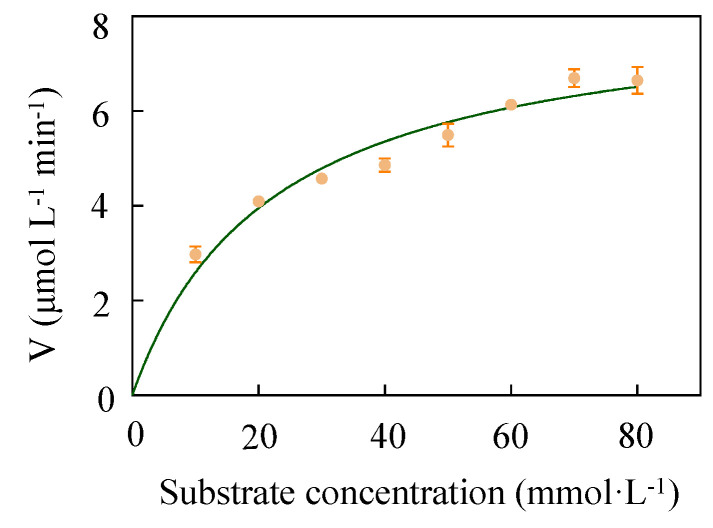
The kinetic parameters of LUCA–DmpA. Initial rates of L-carnosine formation were analyzed in reaction systems containing 100 mM L-His and eight concentrations of β-Alanine methyl ester hydrochloride (10 mM, 20 mM, 30 mM, 40 mM, 50 mM, 60 mM, 70 mM and 80 mM) at optimal temperature (45 °C) and pH (9.0).

In this study, enzyme activity properties (*K*_m_ and *V*_max_) were measured using substrates with different molar ratios. As detailed in Table 1 and Figure 6, *K*_m,_ *k*_cat_ and the *k*_cat_*/K*_m_ of LUCA–DmpA were measured as 22.06 ± 1.02 mmol·L^−1^, 76.53 ± 2.68 s^−1^ and 3.47 s^−1^·L·mmol^−1^, respectively. As a value represents the affinity between the enzyme and the substrate, a higher affinity is suggested by a *K*_m_ with smaller value. With L-His and β-Alanine methyl ester hydrochloride as a substrate, although LUCA–DmpA tended to present a lower affinity than previously reported for β-aminopeptidase BapA (*K*_m_ = 23.00 mM) and DmpA (*K*_m_ = 0.48 mM), LUCA–DmpA showed a higher catalytic efficiency than BapA (*k*_cat_ = 0.87 s^−1^) and DmpA (*k*_cat_ = 12.90 s^−1^), indicating that this predicted ancient enzyme presents excellent substrate conversion ability [20]. Moreover, regarding *k*_cat_*/K*_m_, which is considered to be the most comprehensive index for measuring the catalytic ability of an enzyme, this suggested that the current newly expressed ancient enzyme had a higher catalytic efficiency than BapA, which was identified with a *k*_cat_/*K*_m_ value of 0.04 s^−1^·L·mmol^−1^.

## 3. Conclusions

In this study, the ancestors of four peptidase families (P1, T4, S58 and D-aminopeptidase) were reconstructed using ASR and the screened sequence was further expressed to obtain aminopeptidase in *E. coli* BL21 (DE3) with more desirable properties regarding the enzymatic synthesis function of L-carnosine. Although two unexpected polypeptides were observed due to the autoproteolytic mechanism of LUCA–DmpA, contributed by the potential environmental adaptability of ancestral archaea to harsh living conditions, the current studied enzyme was observed to have improved pH tolerance within a pH range from 5 to 11 and strong resistance capability to hydrophobic organic solvents compared with other reported present-day enzymes. In addition, LUCA–DmpA exerted a relatively high catalytic efficiency in carnosine synthesis (*K*_m_ = 22.06 ± 1.02 mmol·L^−1^, *k*_cat_ = 76.53 ± 2.68 s^−1^, *k*_cat_/*K*_m_ = 3.47 s^−1^·L·mmol^−^^1^). These excellent properties create favorable conditions for L-carnosine synthesis. In addition, by obtaining this ancient enzyme using the ASR, the current study provided a promising pathway of enzymatic production of L-carnosine as well as suggested its potential in industrial applications with regard to peptide synthesis.

## 4. Materials and Methods 

### Ancestral Sequence Reconstruction 

The amino sequences of aminopeptidase used in this study were obtained from the NCBI GenBank databases (http://www.ncbi.nlm.nih.gov, accessed on 15 August 2022). To construct the maximum likelihood phylogenetic tree, multiple sequence alignments of homologous sequences of β-aminopeptidase DmpA (WP_043062538.1), BapA(XP-033415514.1) and 3N5I-A that screened out from NCBI using BLAST were performed first using the MAFFT v7.471 program [31]. Following the selection of the best amino acid substitution model using ProTest v3.4.2, the maximum likelihood phylogenetic tree of β-aminopeptidase was created using PhyML v3.0 [32]. Based on the above, the strictly conserved amino acid residues and the most probable ancestral protein sequences were identified using ESPript 3.0 [33] and ancestral sequence reconstruction was accomplished using the FireProtASR (http://loschmidt.chemi.muni.cz/fireprotasr/, accessed on 20 August 2022) web server [34], respectively.

## Figures and Tables

**Table 1:** The kinetic parameters of LUCA–DmpA, reported BapA and DmpA. Initial rates of L-carnosine formation catalyzed by LUCA–DmpA were analyzed from 100 mM L-His and eight concentrations of β-Alanine methyl ester hydrochloride (10 mM, 20 mM, 30 mM, 40 mM, 50 mM, 60 mM, 70 mM and 80 mM) catalyzed by LUCA–DmpA at 45 °C for 10 min at pH = 9.0.

Kinetic Parameters	LUCA–DmpA	DmpA	BapA
*K*_m_ (mM)	22.06 ± 1.02	0.48 ± 0.05	23.00 ± 8.00
*k*_cat_ (s^−1^)	76.53 ± 2.68	12.90 ± 0.59	0.87 ± 0.23
*k*_cat_/*K*_m_ (s^−1^ L mmol^−1^)	3.47	26.88	0.04

## Data Availability

Supplementary Materials File is available.

## Supplementary Materials

The following supporting information can be downloaded at: https://www.mdpi.com/article/10.3390/molecules27196620/s1. Concluding materials and methods, simplified phylogenetic tree, LC−MS results of L-carnosine and enzymatic product [35]. Figure S1: The simplified phylogenetic tree inferred using Maximum Likelihood method. The tree root indicates the ancestor (LUCA–DmpA) of four peptidase families (T4, P1, S58 and D−aminopeptidase). Figure S2: Constructed pET28a-LUCA-DmpA expression plasmid and its verification result of single and double digestion, respectively. Figure S3: SDS-PAGE analysis of purified LUCA–DmpA only containing N−terminal His−tag. Figure S4: The detection result of LC−MS.
