# Supplementary material for: Preparation and Characterization of an Ancient Aminopeptidase Obtained from Ancestral Sequence Reconstruction for L-Carnosine Synthesis
Source: Molecules. 2022 Oct 5;27(19):6620. doi: 10.3390/molecules27196620 (PMC9570944; doi:10.3390/molecules27196620)
Supplement: Supplementary file 1 [file molecules-27-06620-s001.zip › molecules-1943374-supplementary.pdf]

# Supporting Information

## Preparation and characterization of an ancient aminopeptidase obtained from ancestral sequence reconstruction for L-carnosine synthesis

Fan Liu[a,b], Yi Shi[a,b] , Yakun Fang[a,b], Zhenshan Liu[a,b], Yu Xin[a,b], Zhenghua Gu[a,b], Zitao Guo[a,b], Liang Zhang[a,b]*

[a] *National Engineering Research Center of Cereal Fermentation and Food Biomanufacturing, Jiangnan University, Wuxi 214122, China*

[b] *Jiangsu Provincial Research Center for Bioactive Product Processing Technology, Jiangnan University, Wuxi 214122, China*

**Corresponding author. Email: zhangl@jiangnan.edu.cn (L. Zhang), telephone/fax number: +86 051085918235*

1. **Materials and methods**

*1.1. Bacterial strains and plasmids*

To have the screened amino sequences of aminopeptidase expressed for further enzymatic mechanism investigation, the obtained sequence was codon-optimized firstly before synthesizing and insertion into the plasmid PUC57 by Sangon Biotech (Shanghai, China). Using *E. coli* BL21 (DE3) as the host, the gene encoding ancient enzyme was ligated into the pET-32a, PTIG and pET-28a(+) for protein expression after amplification, respectively. Finally, this 1080 bp gene of ancient enzyme was ligated and expressed in pET-28a(+) vector for the generation of protein with soluble form. The primers listed in Table 2 were designed on the basis of nucleotide sequence for PCR. The primers listed in Table 2 were designed on the basis of nucleotide sequence for PCR.

**Table S1.** Primers for plasmid construction.

| Primer | Sequence (5′ → 3′) |
|---|---|
| AP-F | ACAGCAAATGGGTCGCGGATCCATGAAGCGCGCACGTCTG |
| AP-F | TGCTCGAGTGCGGCCGCAAGCTTTTAACGACGCCCGGGGCGCT |

*1.2. Cloning, expression and purification of β-aminopeptidase (LUCA-DmpA)*

After the recombinant plasmid pET28a(+)-LUCA-DmpA was transformed into *E. coli* BL21 for expression (The recombinant plasmid only containing a N-terminal His-tag.). After that, the positive transformants of BL21 were selected and cultured in 20 mL LB medium including 50 μg·mL$^{-1}$ of

kanamycin at 37°C, 200 rpm for 12 hours, and then the seed culture was subsequently transferred to 50 mL LB medium (50 μg·mL$^{-1}$ kanamycin) at 1.5% inoculum at 37°C, 200 rpm till the optical density at 600 nm reached 0.6-0.8. IPTG was then added to the medium with a final concentration of 0.1 mmol·L$^{-1}$ and the recombinant cells were further cultivated at 16°C for 24 hours.

After the recombinant cells were collected by centrifugation at 4°C, 12000rpm for 2 min, washed twice with Tris-HCl buffer, and disrupted by ultrasonification, the crude enzyme was obtained by centrifugation (4°C, 12000 rpm, 15 min). Purified enzyme was collected with a Histrap HP column. In brief, solution A (50 mM Tris-HCl buffer, 0.5 M sodium chloride, 20 mM imidazole, pH 8.0) and solution B (50 mM Tris-HCl buffer, 0.5 M sodium chloride, 500 mM imidazole, pH 8.0) were used for gradient elution of the unbound protein (50 mM imidazole) and target protein (300 mM imidazole), respectively. Then, the imidazole of the pure recombinant enzymes was removed for further SDS-PAGE.

### *1.3. Western Blot and N-terminal sequencing*

Western Blot was performed for further detection of the target LUCA-DmpA and a recombinant expression vector only having a C-terminal His-tag was reconstructed for analysis. Specifically, the purified recombinant enzyme was transferred from SDS-PAGE gel to PVDF membrane in transfer buffer (1 L: 20 mM Tris, 40 mM Glycine, 200 mL Methanol) at 350 mA for 90 min. Then, the PVDF membrane was blocked for 2 h with 5% milk in TBST (150 mM NaCl, 10 mM Tris, 0.1% Tween 20, pH 8.0). After the first anti-His antibody (1:5000) was incubated for 2 h and the membrane washed by TBST three times for 10 min, the rabbit secondary antibody (1:5000) was applied for 1 h at room temperature. After a final wash of TBST for 30 min (three changes), the results were analyzed with a gel imager (Bio-Rad, USA).

N-terminal sequencing was conducted herein to provide comprehensive understanding of the enzymatic mechanism of the LUCA-DmpA. In detail, the enzymes were transferred from Tricine-SDS-PAGE gel to PVDF membrane in CAPS buffer and the other electroporation conditions were in accordance with that executed for western blot. The PVDF membrane was stained with Ponceau S Staining Solution for 15 min and washed by deionized water till the target protein band was clear. The 15 kDa protein band was excised and then submitted to Shanghai Applied Protein Technology for N-terminal amino acids sequencing (PPSQ53A, Shimadzu).

### *1.4. Physical property and structural characterization*

The denaturation temperature ($T_m$) of purified LUCA-DmpA was measured by Nano-differential scanning calorimetry (Nano-DSC) (Waters, USA). Before measurement, the sample was measured three times with phosphate buffer (20 mM, pH 9.0). The purified recombination enzyme was then scanned by increasing the temperature from 20°C to 80°C at a heating rate of 1°C·min$^{-1}$[1]. The experimental results were analyzed by Nano Analyze software.

The proportion of each secondary structure in purified aminopeptidase was estimated by circular dichroism (CD) spectrum. After the 1 mm cuvette was rinsed by phosphate buffer, the CD spectrum of

purified enzyme (0.2 mg·mL$^{-1}$) was scanned with a MOS-450 AF spectropolarimeter (BioLogic, France) from 190 to 250 nm. The results are the average of three measurements.

*1.5. Enzymatic L-carnosine Synthetization Evaluation*

Analyzing by High Performance Liquid Chromatography (HPLC), the performance of the obtained LUCA-DmpA for the enzymatic synthesis of L-carnosine was evaluated by having the reaction performed in 5 mL phosphate buffer (20 mM, pH = 9) containing 100 mM L-His and 50 mM β-Alanine methyl ester hydrochloride. The reaction was started by adding 100 μL (0.9 mg·mL$^{-1}$) purified enzymes and then processed at 25°C for 10 min. 1 M HCl was used for the termination of reaction by adjusting the reacting system to the pH of 4.0. The catalytic activity was then evaluated by the measurement of the final concentration of L-carnosine with HPLC. One unit (U) of the enzyme activity was defined as the amount of enzyme that catalysis the synthesis of 1 μmol L-carnosine per minute. The mixture system that without enzyme was applied at the blank. The enzyme activity of ancient enzyme was calculated by Equation (1).

$$U = \frac{CV}{T} \qquad \text{Eq (1)}$$

Where U (μmol·min$^{-1}$) represents the activity of LUCA-DmpA, C (μM) is the concentration of L-carnosine, V (mL) is the total volume of reaction system, T (min) is the reaction time.

*1.6. Effects of pH and temperature on enzyme activity and stability*

Maintained at the same experimental conditions described in Sec. 3.6, LUCA-DmpA enzymatic activity was measured against a temperature range of 15-60°C. With the maximum activity as control (100%), the relative enzyme activity at other temperatures was measured to determine the optimal temperature of LUCA-DmpA. The enzymatic thermostability was investigated by determining the residual enzyme activity after an incubation at several temperatures (30, 35, 40, 45, 50, 55, 60 and 65°C) for 30 min.

The optimal pH of LUCA-DmpA was assessed in the pH range of 4 - 11 at various pH buffers, which including acetate buffer (pH 4.0 - 5.0), phosphate buffer (pH 6.0 - 7.0), Tris-HCl buffer (pH 8.0 - 9.0) and glycine-sodium hydroxide buffer (pH 10.0 - 11.0). Regarding pH stability, it was identified by measuring the remaining enzyme activity after the incubation of enzyme at different pH buffers at room temperature for 12 h with the maximum activity as control (100%).

*1.7. Effect of different organic solvents on enzyme activity*

To evaluate whether there is an impact of organic solvents on the enzyme activity of this newly screened out aminopeptidase, different organic substances (n-hexane, toluene, tert-butanol, isopropanol, ethanol, acetonitrile, methanol and DMSO) with a variation in log P value was used herein. In detail, the enzyme was incubated within these different organic solvents (50%, v/v) at room temperature for 30 min, while the reaction was further processed by having the mixture incubated at 45°C for 10 min. The reaction system without organic solvent was set at the control (100%).

*1.8. Effect of metal ions*

The effects of different kinds of metal ions ($Al^{3+}$, $Ba^{2+}$, $Mn^{2+}$, $K^{+}$, $Zn^{2+}$, $Co^{2+}$, $Ca^{2+}$, $Mg^{2+}$, $Fe^{3+}$ and $Cu^{2+}$) on enzyme activity were measured at three different concentrations (0.5 mM, 1.0 mM, 3.0 mM). With the reaction system without metal ions as control (100%), the activity was then determined by having the above metal ions added respectively to the reaction mixture under experimental conditions described above.

*1.9. The Kinetic parameters of LUCA-DmpA*

For the determination of the kinetic parameters of the LUCA-DmpA, the concentration of L-His was kept constant at 100 mM and the enzyme activity assays were measured at eight concentrations (10 mM, 20 mM, 30 mM, 40 mM,50 mM,60 mM,70 mM,80 mM) of β-Alanine methyl ester hydrochloride. The protein concentration of ancient enzyme was 0.76 $mg \cdot mL^{-1}$. The initial velocity of the reaction was tested by having the reaction mixture at 45˚C for 10 min. The Michaelis–Menten's constant ($K_m$) and $V_{max}$ ($V_m$) were calculated through nonlinear curve and date was analyzed by prism software (GraphPad Software, USA). The $k_{cat}$ and $k_{cat}/K_m$ were calculated by Equation (2) and (3) to determine the affinity of the ancient enzyme for its substrate.

$$k_{cat} = \frac{V_m}{[E] \cdot T} \qquad \text{Eq (2)}$$

$$[E] = \frac{C \cdot V}{M} \qquad \text{Eq (3)}$$

Where $k_{cat}$ ($s^{-1}$) is catalytic number, $V_m$ ($\mu mol \cdot min^{-1}$) is the maximum reaction rate, [E] (μmol) is the amount of active center of ancient protein, T (s) is the reaction time, c ($mg \cdot mL^{-1}$) is the protein concentration of ancient enzyme, V (μL) is the enzyme reaction volume, M (Da) is the molecular mass of LUCA-DmpA.

*1.10. Statistical analysis*

Data were expressed as mean ± standard deviation from three parallels per trial.

## 2. Figures

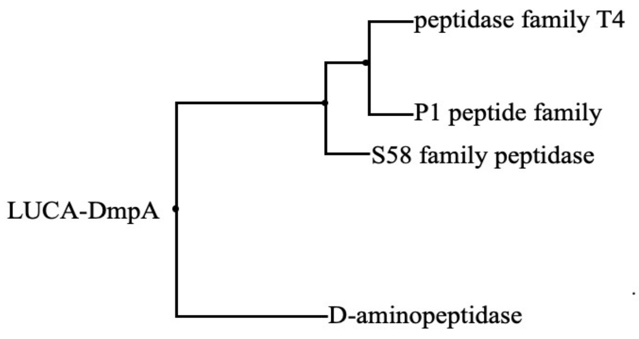


**Figure S1.** The simplified phylogenetic tree inferred using Maximum Likelihood method. The tree root indicates the ancestor (LUCA-DmpA) of four peptidase families (T4, P1, S58 and D-aminopeptidase)

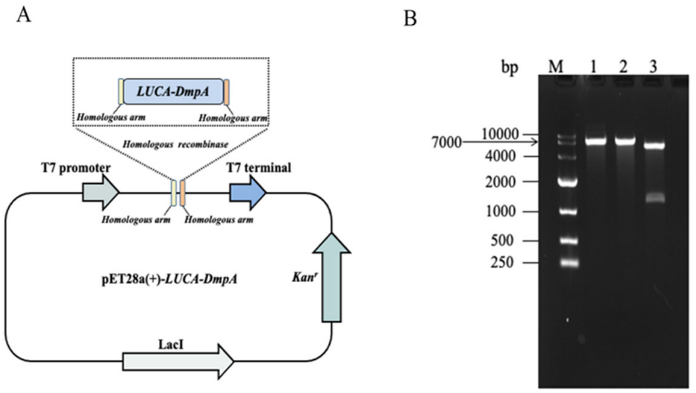


**Figure S2.** Constructed pET28a-LUCA-DmpA expression plasmid and its verification result of single and double digestion, respectively. A) LUCA-DmpA gene was ligated into pET-28a (+) vector using homologous recombinase. B) the plasmid was verified by single digestion and double digestion. Lane M, the molecular marker; lane 1, *BamH I* single digestion; lane 2, *Hind III* single digestion; lane 3, *BamH I* and *Hind III* double digestion.

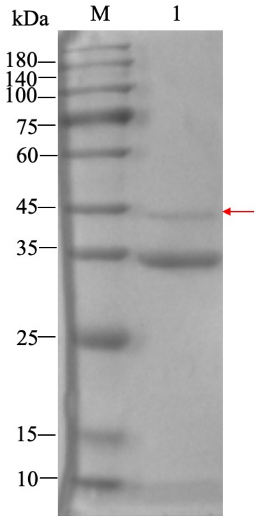


**Figure S3.** SDS-PAGE analysis of purified LUCA-DmpA only containing N-terminal His-tag.

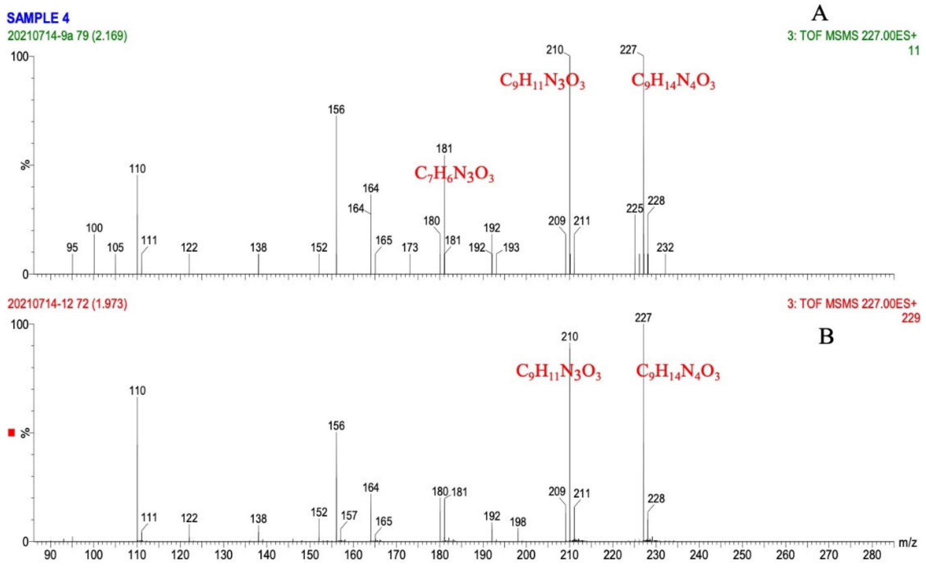

**Figure S4.** The detection result of LC-MS. A) The LC-MS result of enzymatic reaction product. B) The LC-MS result of L-carnosine.

## 3. The gene sequence of LUCA-DmpA

ATGAAGCGTGCCCGTCTGCGTGATCTGGGCATTACCATTGGCCGCCTGCCGACAGGTCCGTATAATGCTATTA
CCGATGTTCCGGGTGTGCGTGTGGGTCATACCACCATTATTGAAGATGATCCGCATGTGGTTAACACCGGCGT
TACCACCATTCTGCCGCAGGATGGCGAAGTTTGGGAACATCATGTTTTTGCCGGTTATTTTCGTTTCAACGGT
AGCGGTGAAATGACCGGCAGTCATTGGCTGGAAGAAAGTGGCCTGCTGAGTAGTCCGGTGATTATTACCAAT
AGCTTTGGCGTGGGCGCATGTTATGATGCACTGGTTAAATATGCCGCAGAACAGGACCCTACCGCACCGTTTA
CCCTGCCTGTGATTGCCGAAACCTTTGATGGCTGGCTGAGCGATATTGGTGCAATGGCTGTGACCCCGGAAC
ATGTTCGTGAAGCCCTGGAAAATGCCCGCAGTGGTCCGGTTGCAGAAGGTAATGTGGGTGGCGGCACCGGA
ATGATTACAATGGGTTTTAAAGCAGGCACCGGCACCAGTAGCCGCGTTGTGGAGGTTGAAGGCGAAGGTTAT
ACCGTTGGTGCCCTGGTGCAGAGCAATTTTGGCGGTGCCCGCTTTCTGACCATTAATGGTGTTCCGGTTGGCC
GTCTGATTCCGGCAGATCAGGTTCCGGTGCCGTGGGAGGAACCTCCTAGAGAAGATGGTAGCATTATTGTTAT
CATCGCAACCGATGCCCCGCTGCTGCCTCATCAATGCAAACGTCTGGCCCGTCGTGCCACCTTAGGTGTTGC
ACGTACCGGCGGTTGGGGTAGTAATTATAGCGGCGATATTTTTCTGGCCTTTAGTACCGGCAATCGCCTGCCG
CGTCAGCCTGAGGAGCCTGTGTATGGTCTGAAAATGCTGCCGAATGAAGAAATGGACCCTCTGTTTCAGGGC
GCCGTTGAAGCAACCGAAGAAGCCATTCTGAATGCCATTTGCATGGCCGAAACCATGAAAGGCCGCAAAGG
CCGTGAAGTGAAAGCACTGCCGCTGGATCGCCTGAAAGAAATTCTGAAACGTCCGGGCCGCCGCTAA

# References


1. Xin, Y.; Zheng, M.; Wang, Q.; Lu, L.; Zhang, L.; Tong, Y.; Wang, W. Structural and catalytic alteration of sarcosine oxidase through reconstruction with coenzyme-like ligands. Journal of Molecular Catalysis B: Enzymatic 2016, 133, S250-S258.
